# Crimean-Congo Hemorrhagic Fever, Southwestern Bulgaria

**DOI:** 10.3201/eid1506.081567

**Published:** 2009-06

**Authors:** Iva Christova, Antonino Di Caro, Anna Papa, Concetta Castilletti, Lubena Andonova, Nikolay Kalvatchev, Evangelia Papadimitriou, Fabrizio Carletti, Emad Mohareb, Maria R. Capobianchi, Giuseppe Ippolito, Giovanni Rezza

**Affiliations:** National Centre of Infectious and Parasitic Diseases, Sofia, Bulgaria (I. Christova, N. Kalvachev); National Institute for Infectious Diseases “L. Spallanzani,” Rome, Italy (A. Di Caro, C. Castilletti, F. Carletti, M.R. Capobianchi, G. Ippolito); Aristotelian University of Thessaloniki, Thessaloniki, Greece (A. Papa, E. Papadimitriou); Infectious Diseases Hospital, Sofia (L. Andonova); US Naval Medical Research Unit 3, Cairo, Egypt (E. Mohareb); Istituto Superiore di Sanità, Rome (G. Rezza).

**Keywords:** Viruses, Crimean-Congo hemorrhagic fever, Crimean-Congo hemorrhagic fever virus, PCR, cluster, Bulgaria, letter

**To the Editor**: Crimean-Congo hemorrhagic fever virus (CCHFV) causes a severe multisystem disease characterized by profuse bleeding with a case-fatality rate as high as 30%. The infection is endemic to the Balkans (*1*,*2*). In Bulgaria, most cases are reported from the central and eastern parts of the country (*3*,*4*). We report a cluster of cases observed in early spring 2008 in southwestern Bulgaria, an area considered at low risk for CCHF outbreaks.

The index case-patient was a 49-year-old man in whom fever, severe myalgia and joint pain, diarrhea for 1 day, cough, and weakness developed on March 20. Three days before, while not using hand protection, he removed ticks from cows. On March 25, severe epistaxis developed and he was hospitalized. His condition rapidly deteriorated; leukopenia, thrombocytopenia, and elevated levels of liver enzymes developed, and he died on March 26. The autopsy found hemorrhages in the lungs but not in the hypophysis or gastrointestinal tract. Immunoglobulin (Ig) M antibodies against CCHFV were detected in the serum sample.

The second case-patient was a 34-year-old man who had removed ticks from cows from the same herd as the index case-patient. Symptoms developed on March 23 and he was hospitalized on March 26 with fever, diarrhea, and bloody sputum. Laboratory findings showed moderate leukopenia and thrombocytopenia. His condition improved within 1 week. IgM antibodies against CCHFV were detected in a serum sample collected during the acute phase of the disease.

The third confirmed case-patient was a 52-year-old woman (nurse) who cared for the index case-patient after his hospital admission. Although she reported wearing gloves, she was extensively exposed to the patient’s blood and vomit and received immunoprophylaxis (specific hyperimmune gamma globulins). On March 28, a mild disease characterized by fever, headache, weakness, and maculopapular rash with petechiae developed; she was hospitalized on April 2. She had leukopenia, thrombocytopenia, and normal levels of liver enzymes. The serum sample collected during the acute phase of the disease was IgM positive, and a 4-fold increase was present in the IgG titer in a sample collected during the convalescent phase (from 160 to 640). Blood and serum samples taken during the acute phase of the disease were positive for CCHFV by real-time PCR (*5*) and reverse transcription–nested PCR (*6*). Purified PCR product was sequenced; the nucleotide sequence was submitted to GenBank (accession no. FJ160262). Viral load was 3.88 × 10^7^ copies/mL.

The fourth confirmed case-patient was a 50-year-old woman, the wife of the index case-patient. She was hospitalized April 10 with fever, headache, myalgia, weakness, stomach pain, and nausea. She reported exposure to her husband’s blood before hospital admission. Thus, hyperimmune gamma globulins against CCHFV were administered. She had leukopenia, thrombocytopenia, and elevated levels of aspartate aminotransferase and alanine aminotransferase. The symptoms lasted only 7 days. CCHFV was detected by both PCRs (*5*,*6*) in a serum sample taken on day 3 of the disease; sequence of the PCR products was submitted to GenBank (accession no. FJ445749).

A phylogenetic tree including sequences from the third and fourth cases was constructed (Figure). The 2 sequences clustered within the Europe/Turkey clade. The genetic distance between the 2 strains was 1.15%, but the 2 sequences were identical at the amino acid level. Sequences from the present study showed 96.4%–98.8% similarity with respective CCHFV sequences from Bulgaria from a former study (BUL10/02 and BUL1/03) (*3*) but differed from the Kosovo 9553/2001 strain by 0.8%–2.0% and from the Greek 66/08 strain by 1.2%–2.4%.

**Figure:**
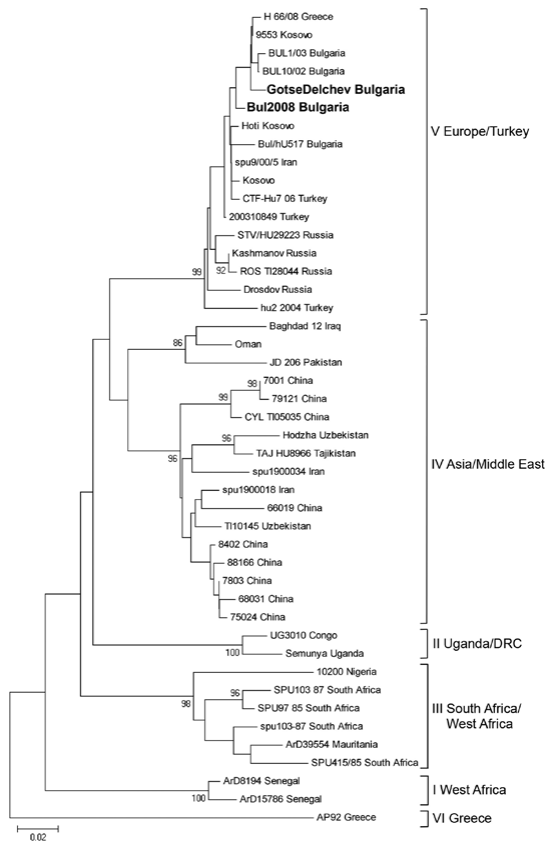
Phylogenetic tree of partial sequence (256 bp) of Crimean-Congo hemorrhagic fever (CCHF) virus nucleoprotein gene. CCHF virus sequences are listed as viral strain name and country of origin. Sequence of case 3 is designated in the tree as “Gotse Delchev Bulgaria,” and sequence of case 4 is designated as “Bul 2008 Bulgaria” (in **boldface**). Strain AP92, Greece, was used as an outgroup. Numbers at the nodes represent bootstrap values. Scale bar indicates number of nucleotide substitutions per site.

Two additional suspected CCHF cases occurred in the same area, on March 30 and April 9 (*7*). Both persons were negative for CCHFV infection. All 119 ticks of various species (*Hyalomma marginatum*, *Dermacentor marginatus*, *Rhipicephalus bursa*, *Ixodes ricinus*) collected from the area and tested by reverse transcription–nested PCR were negative for CCHFV.

This cluster of CCHF cases has several important highlights. First, it occurred in a region that was considered to have low CCHF endemicity; however, the area is only a few kilometers from Greece, where a human fatal case was observed in June 2008 (*8*). The index case was observed earlier in the year than in previous years, and clinical manifestations of the cases were unusual (absence of craniopharyngeal syndrome and bleeding from gastrointestinal tract that are typical for CCHF patients from Bulgaria); in the fatal case, autopsy of the patient showed hemorrhages only in the lungs. Two cases were attributable to tick exposure, whereas the other 2 were most likely secondary cases attributable to contact with the index case-patient (in this regard, CCHFV sequences of the secondary cases were almost identical). Finally, the longer incubation period of the wife of the index case-patient might be associated with administration of hyperimmune gamma globulin against CCHFV.

In conclusion, CCHF emerged in southwestern Bulgaria near the border with Greece. Person-to-person transmission emphasizes the need for rapid diagnosis of CCHF, especially in cases with atypical clinical manifestations.
